# Network Structure Explains the Impact of Attitudes on Voting Decisions

**DOI:** 10.1038/s41598-017-05048-y

**Published:** 2017-07-07

**Authors:** Jonas Dalege, Denny Borsboom, Frenk van Harreveld, Lourens J. Waldorp, Han L. J. van der Maas

**Affiliations:** Department of Psychology, University of Amsterdam, 1018 WT Amsterdam, The Netherlands

## Abstract

Attitudes can have a profound impact on socially relevant behaviours, such as voting. However, this effect is not uniform across situations or individuals, and it is at present difficult to predict whether attitudes will predict behaviour in any given circumstance. Using a network model, we demonstrate that (a) more strongly connected attitude networks have a stronger impact on behaviour, and (b) within any given attitude network, the most central attitude elements have the strongest impact. We test these hypotheses using data on voting and attitudes toward presidential candidates in the US presidential elections from 1980 to 2012. These analyses confirm that the predictive value of attitude networks depends almost entirely on their level of connectivity, with more central attitude elements having stronger impact. The impact of attitudes on voting behaviour can thus be reliably determined before elections take place by using network analyses.

## Introduction

Suppose you are one of the more than 130 million Americans who voted in the presidential election in 2016. Let us further assume that you were supportive of Hillary Clinton: You mostly held positive beliefs (e.g., you thought she was a good leader and a knowledgeable person) and you had positive feelings toward her (e.g., she made you feel hopeful and proud), representing a positive attitude toward Hillary Clinton^1–4^. However, you also held a few negative beliefs toward her (e.g., you thought that Hillary Clinton was not very honest). Did your overall positive attitude cause you to vote for Hillary Clinton? Here we show that the answer to this question depends on the network structure of your attitude: First, we show that the impact of attitudes (i.e., average of the attitude elements) on behavioural decisions depends on the connectivity of the attitude network (e.g., the network of your positive attitude toward Hillary Clinton was highly connected, so you probably voted for Hillary Clinton). Second, we show that central attitude elements have a stronger impact on behavioural decisions than peripheral attitude elements (e.g., your positive beliefs about Hillary Clinton were more central in your attitude network than your negative beliefs, so the chance that you voted for Hillary Clinton further increased). We thus provide insight into how structural properties of attitudes determine the extent to which attitudes cause behaviour.

**Figure 1:**
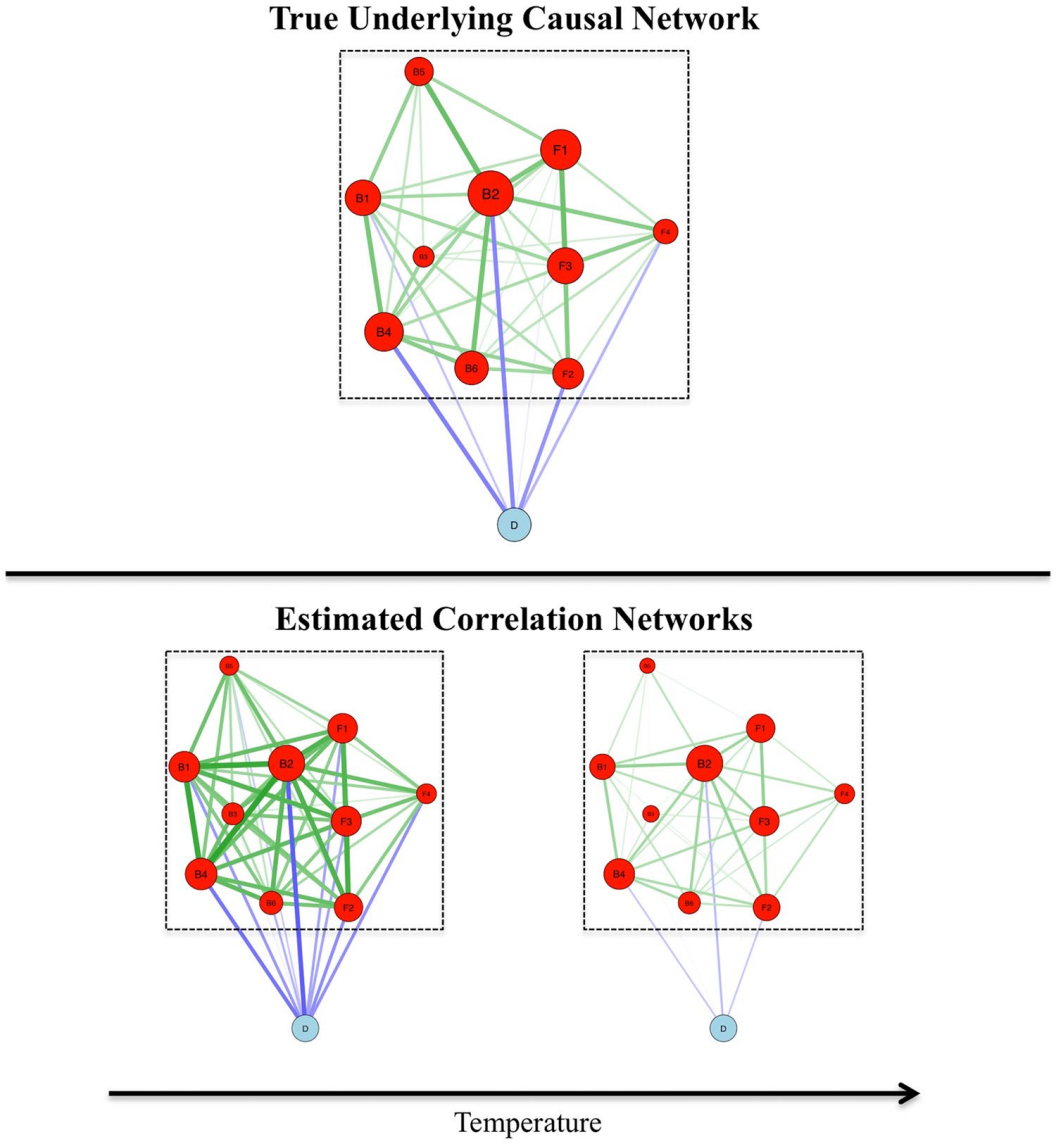
Illustrations of the Causal Attitude Network model and the hypotheses of the current study. Networks represent a hypothetical attitude network toward a presidential candidate consisting of six beliefs (e.g., judging the candidate as honest, intelligent, caring; represented by nodes B1 to B6), four feelings (e.g., feeling hope, anger toward the candidate; represented by nodes F1 to F4), and the voting decision (represented by the node D). Red nodes within the dashed square represent the part of the network on which connectivity and centrality estimates are calculated. Edges represent positive bidirectional causal influences (correlations) in the causal network (correlation networks), with thicker edges representing higher influence (correlations). Note that in this network, we assume that positive (negative) states of all nodes indicate a positive (negative) evaluation (e.g., positive state of judging a candidate as honest [dishonest] would be to [not] endorse this judgment). Size of the red nodes corresponds to their closeness centrality (see Methods for details on the network descriptives). In the CAN model, temperature represents a formalized conceptualization of consistency pressure on attitude networks. The correlation networks illustrate that lower (higher) temperature implies higher (lower) correlations between the attitude elements.

In network theory, dynamical systems are modelled as a set of nodes, representing autonomous entities, and edges, representing interactions between the nodes^5^. The set of nodes and edges jointly defines a network structure. Modelling complex systems in this way has probably become the most promising data-analytic tool to tackle complexity in many fields^6^, such as physics^7, 8^, biology^9^, and psychology^10–13^. Recently, network analysis has also been introduced to the research on attitudes in the form of the Causal Attitude Network (CAN) model^1^. In this model, attitudes are conceptualized as networks, in which nodes represent attitude elements that are connected by direct causal interactions (see Fig. 1). The CAN model further assumes that the Ising model^14^, which originated from statistical physics, represents an idealized model of attitude dynamics.

In the Ising model, the probability of configurations (i.e., the states of all nodes in the network), which represents the overall state of the attitude network, depends on the amount of *energy* of a given configuration. The energy of a given configuration can be calculated using the Hamiltonian function:

1 $$H(x)=-\sum _{i}{\tau }_{i}{x}_{i}-\sum _{\langle i,j\rangle }{\omega }_{i}{x}_{i}{x}_{j}.$$

Here, *k* distinct attitude elements 1,…,*i*, *j*,….*k* are represented as nodes that engage in pairwise interactions; the variables *x*_*i*_ and *x*_*j*_ represent the states of nodes *i* and *j* respectively. The model is designed to represent the probability of these states as a function of a number of parameters that encode the network structure. The parameter *τ*_*i*_ is the threshold of node *i*, which determines the disposition of that node to be in a positive state (1; endorsing an attitude element) or negative state (−1, not endorsing an attitude element) regardless of the state of the other nodes in the network (statistically, this parameter functions as an intercept). The parameter *ω*_*i*_ represents the edge weight (i.e., the strength of interaction) between nodes *i* and *j*. As can be seen in this equation, the Hamiltonian energy decreases if nodes are in a state that is congruent with their threshold and when two nodes having positive (negative) edge weights ﻿between them﻿ assume the same (different) state. Assuming that attitude elements of the same (different) valence are generally positively (negatively) connected, attitude networks thus strive for a consistent representation of the attitude. The probability of a given configuration can be calculated using the Gibbs distribution^15^:

2 $$Pr(X=x)=\frac{\exp (-\beta H(x))}{Z},$$

in which *β* represents the inverse temperature of the system, which can be seen as consistency pressures on attitude networks: reducing (increasing) the temperature of the system results in stronger (weaker) influence of the thresholds and weights, thereby scaling the entropy of the Ising network model^16, 17^. An Ising model with low (high) temperature results in a highly (weakly) connected correlation network (see Fig. 1). The denominator *Z* represents the sum of the energies of all possible configurations, which acts as a normalizing factor to ensure that the sum of the probabilities adds up to 1.

Conceptualizing attitudes as Ising models allows for the derivation of several hypotheses and a crucial test of this conceptualization is whether it can advance the understanding of the relation between attitudes and behavioural decisions. In the present paper we apply the CAN model and are the first to (a) formalize and (b) test hypotheses based on the CAN model regarding the impact of attitudes on behaviour.

The impact of attitudes on behaviour has been one of the central research themes in Social Psychology in recent decades^18–20^. The bulk of the research on the relation between attitudes and behaviour has been done under the umbrella definition of attitude strength, which holds that one central feature of strong attitudes is that they have a strong impact on behaviour^21^. Several lines of research have identified factors related to attitude strength. Among the most widely researched of these are attitude accessibility, attitude importance, and attitudinal ambivalence. Studies have shown that accessible attitudes (i.e., attitudes that can be easily retrieved from memory) have more impact on behaviour^19, 22^. Similarly, higher levels of (subjective) attitude importance (i.e., attitudes, to which a person attaches subjective importance) are related to increased accessibility of attitudes^23^ and to higher levels of consistency between attitudes and behaviour^24, 25^. Ambivalent attitudes (i.e., attitudes that are based on both negative and positive associations) are less predictive of behaviour than univalent attitudes^26, 27^. While these and other attitude strength attributes, such as certainty and extremity, are generally interrelated^28, 29^, a framework that unifies these different attributes has long been absent in the literature. Recently, however, based on the development of the CAN model, attitude strength was formally conceptualized as network connectivity^1^. The CAN model might thus provide the basis for a comprehensive and formalized framework of the relationship between attitudes and behaviour. Our current aim is to develop and test such a framework. To do so, we first formally derive hypotheses regarding the impact of attitudes on behaviour from the CAN model. Second, we test these hypotheses in the context of voting decisions in the US American presidential elections.

From the CAN model the hypothesis follows that highly connected attitude networks (i.e., attitude networks that are based on Ising models with low temperature) have a strong impact on behaviour. As can be seen in Fig. 1, low temperature results in strong connections both between non-behavioural attitude elements (i.e., beliefs and feelings) *and* between non-behavioural attitude elements and behaviours (e.g., behavioural decisions)^1^. Attitude elements in highly connected networks are thus expected to have a strong impact on behavioural decisions. This leads to the hypothesis that the overall impact of attitudes depends on the connectivity of the attitude network. While the connectivity of attitude networks provides a novel formalization of attitude strength, earlier approaches to understanding the structure of attitudes fit very well within this framework. For example, studies have shown that important attitudes are more coherent than unimportant attitudes^30, 31^ and that strong attitudes have a more consistent structure between feelings and beliefs than weak attitudes^32^. Also, Phillip E. Converse’s distinction between attitudes and nonattitudes based on stability of responses^33^ relates to our connectivity framework^1^.

In addition to predicting the overall impact of an attitude from the connectivity of the attitude network, the CAN model predicts that the specific impact of attitude elements depends on their centrality (as defined by their closeness). Closeness refers to how strongly a given node is connected both directly and indirectly to all other nodes in the network^34, 35^. In contrast to connectivity, which represents a measure of the whole network, centrality is a measure that applies to individual nodes within the network. Attitude elements high in closeness are good proxies of the overall state of the attitude network, as they hold more information about the rest of the network than peripheral attitude elements, rendering closeness the optimal measure of centrality for our current purposes. We therefore expect central attitude elements to have a stronger impact (directly or indirectly) on a behavioural decision no matter which attitude elements are direct causes of this decision. This can also be seen in Fig. 1, as there is a strong relation between a given node’s centrality and it’s correlation with the behavioural decision. It is important to note here that centrality of attitude elements does not refer to the classical definition of attitude centrality, but to the network analytical meaning of centrality. Specific impact of attitude elements has received somewhat less attention in the attitude literature than the global impact of attitudes, with studies either focusing on the primacy of feelings or beliefs in determining behaviour^36–38^ or on the subjective importance of attitude elements^39, 40^ and these different lines of research have been carried out much in isolation from each other and from the attitude strength research paradigm (for an exception see ref. 39). It is our view that an advantage of the approach we take in this article is that our framework holds promise in unifying these different approaches to understanding the relation between attitudes and behaviour.

In this paper, we first show that the hypotheses put forward here above directly follow from conceptualizing attitudes as networks with a simulation study. We then test these hypotheses using data on attitudes toward candidates and voting in the American presidential elections from 1980–2012. In doing so, we test whether the CAN model provides a comprehensive framework on whether attitudes and which attitude elements drive behavioural decisions. Voting decisions are a perfect test of this postulate, because political attitudes often but not always drive voting decisions^22, 36, 37, 41, 42^.

## Results

### Simulation Study

To show that the hypotheses presented above directly follow from conceptualizing attitudes as networks, we simulated networks using three popular algorithms to generate networks: preferential attachment^7, 43^, small-world network model^8^, and random Erdos-Rényi networks^44^ (see also Supplementary Note 1 for analytical solutions). The networks consisted of 11 nodes (which corresponds to the number of nodes in the empirically estimated networks described below), with ten randomly chosen nodes representing attitude elements and one randomly chosen node representing the behavioural decision. Note that in such small networks, network properties other than density and magnitude of edge weights do not play a fundamental role in determining outcomes of the network.

The simulation of networks followed four steps: First, we created a ‘base’ network using one of the three algorithms. Second, we added edge weights to the base network, either drawn from a normal distribution, a Pareto power law distribution, or a uniform distribution. Third, to simulate responses of individuals holding attitudes with the network structure of the base network, we used the Ising network model^14^. We created 20 different variations of the weighted base network in which the temperature of the Ising model was varied. Fourth, we simulated 1000 individuals based on the variations of the base network. As can be seen in Fig. 1, increasing (decreasing) the temperature results in decreasing (increasing) edge weights in the correlation networks.

We repeated this procedure 100 times for each combination of network generating algorithms and edge weights distributions. To investigate whether simulated attitude elements in highly connected networks (i.e., networks, for which the temperature parameter was low) collectively have a strong impact on the simulated decision, we estimated the global connectivity, defined by the Average Shortest Path Length (ASPL)^45^ of the simulated attitude elements. Note that a low ASPL indicates high connectivity. We correlated the global connectivity with the average impact (which we operationalize as the biserial correlation between the sum score of the simulated attitude elements and the simulated decision) for each set of 20 networks. This resulted in strong negative correlations collapsed over all combinations of network-generating algorithms and edge weights distributions (Pearson correlations: mean *r* = −0.91, s.d. *r* = 0.06) and we found strong negative correlations for all of these combinations (see Supplementary Table 1). To investigate whether central nodes (based on closeness) have a strong impact on a decision, we estimated the centrality of the simulated attitude elements and correlated the centrality estimates with the impact of the simulated attitude elements (which we operationalize as the tetrachoric correlation between a given simulated attitude element and the simulated decision). To exclude the possibility that results are driven by differences in average centrality and impact, we standardized both centrality and impact for each network. This resulted in strong positive correlations in the different sets of attitude networks collapsed over all combinations of network-generating algorithms and edge weights distributions (Pearson correlations: mean *r* = 0.59, s.d. *r* = 0.29) and we found strong positive correlations for all of these combinations (see Supplementary Table 1).

### Test of Connectivity Hypothesis

These simulations show clearly that the CAN model predicts a strong relation between network connectivity (node centrality) and the predictive utility of attitudes (attitude elements) in forecasting behaviour. This confirms that these intuitively derived hypotheses are indeed formal predictions that must follow if the CAN model is a valid model of attitudes. To provide an empirical test of the hypotheses put forward here, we analysed open-access data from the American National Election Studies (ANES) on the US presidential elections from 1980–2012 (total *n* = 16,988). In each ANES between ten and 24 attitude elements were assessed and we selected ten attitude elements for each election that were most similar to each other, see Table 1. On these ten attitude elements, we estimated attitude networks for each of the two (three) main candidates for the elections in 1984–1992 and in 2000–2012 (in 1980 and 1996). This gave us 20 attitude networks in total. Nodes in these networks represent attitude elements toward the given presidential candidate that were rated by the participants. Edges between the nodes represent zero-order polychoric correlations between the attitude elements. Note that because our networks are based on zero-order correlations, these networks only vary in magnitudes of edge weights and not in density, because correlation networks are always fully connected.

**Table 1:** Included attitude elements.

Attitude element	Included in data set	Substituted by
“is honest”*	1988–1996, 2008–2012	“is dishonest”* (1980, 2000–2004), “is decent”* (1984)
“is intelligent”*	1984–1992, 1996 (Clinton), 2000–2012	“is weak”* (1980), “gets things done”* (1996 Dole)
“is knowledgeable”*	1980–2012	NA
“is moral”*	1980–2012	NA
“really cares about people like you”*	1984–2012	“is inspiring”* (1980)
“would provide strong leadership”*	1980–2012	NA
“angry”^†^	1980–2012	NA
“afraid”^†^	1980–2012	NA
“hopeful”^†^	1980–2012	NA
“proud”^†^	1980–2012	NA

*Denotes items tapping beliefs. Participants rated to which extent they agreed that these statements described the candidates.

^†^Denotes items tapping feelings. Participants rated whether the candidates ever made them feel these feelings.

**Figure 2:**
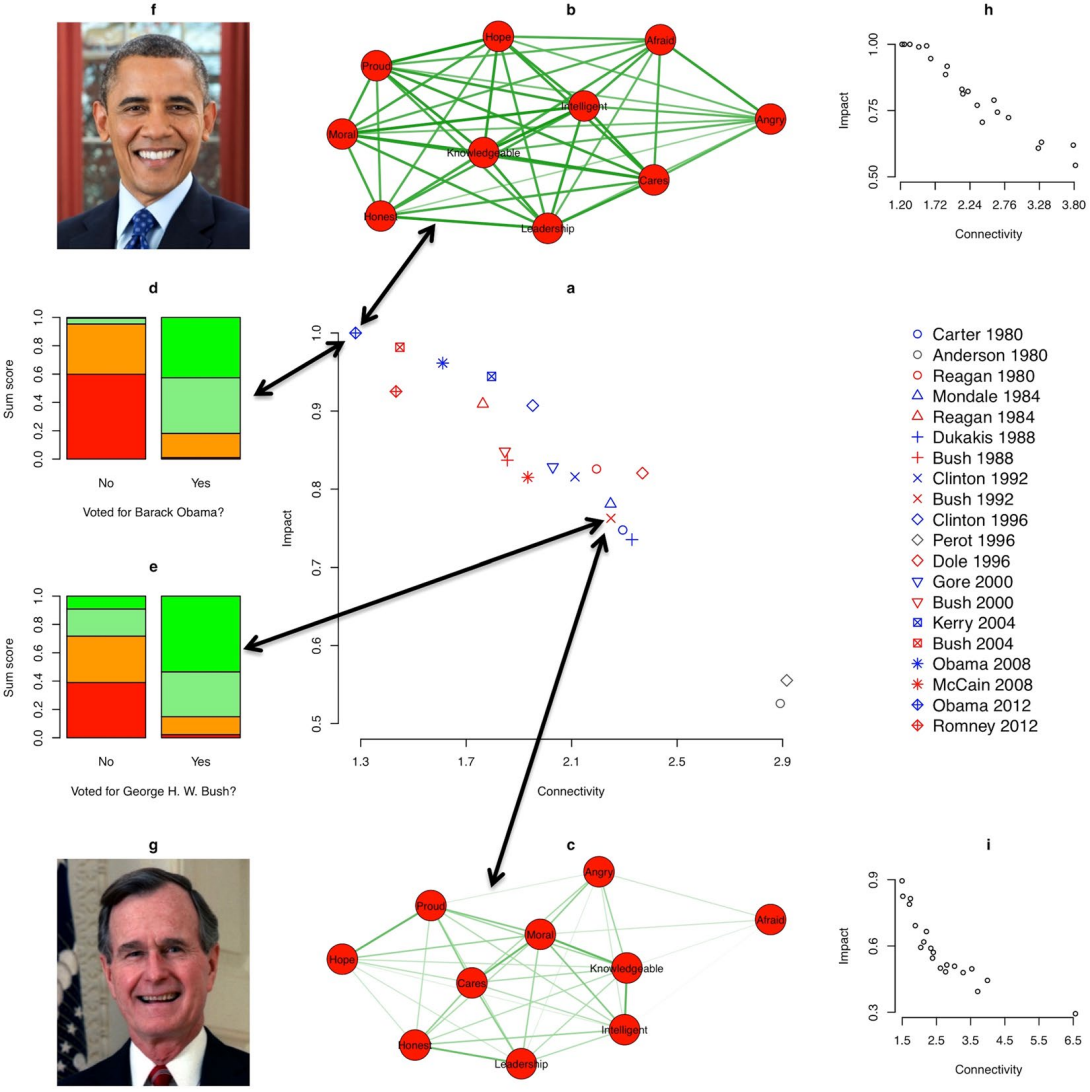
Highly connected attitude networks have a stronger impact on voting decisions than weakly connected attitude networks. (**a**) Relation between connectivity and average impact of attitude elements. (**b**–**e**) Two illustrations of the analytic strategy to assess connectivity and average impact. (**b**,**c**) Attitude network toward Barack Obama (George H. W. Bush) in 2012 (1992). Nodes represent attitude elements and edges represent correlations between attitude elements (the higher the correlation, the thicker the edge; correlations lower than 0.3 are not displayed). Closely connected attitude elements are placed near to each other^70^. (**d**,**e**) Relation between the sum score of attitude elements toward Barack Obama (George H. W. Bush) and voting for Barack Obama (George H. W. Bush). Colours of the bars represent the percentage of individuals who’s sum scores fall into a given percentile (the more green, the higher the sum score; the more red, the lower the sum score). (**f**) Photo of Barack Obama by Pete Souza. Photo is under the CC0/Public Domain Licence. Source: https://www.goodfreephotos.com/people/barack-obama-portrait-photo.jpg.php. (**g**) Photo of George H. W. Bush by unknown photographer. Photo is under the CC0/Public Doman Licence. Source: https://www.goodfreephotos.com/people/george-bush-portrait-photo.jpg.php. (**h**,**i**) Relation between connectivity and impact for the simulated set of networks that was closest to the mean correlation plus (minus) one standard deviation.

First, we tested whether highly connected attitude networks have strong average impact. As in the simulation study, connectivity was based on the ASPL and average impact was operationalized as the biserial correlation between the sum score of attitude elements and the voting decision. As can be seen in Fig. 2, we found a high negative correlation between connectivity and average impact (Pearson correlation: *r* = −0.95, *P* < 0.001), supporting our hypothesis.

### Test of Centrality Hypothesis

**Figure 3:**
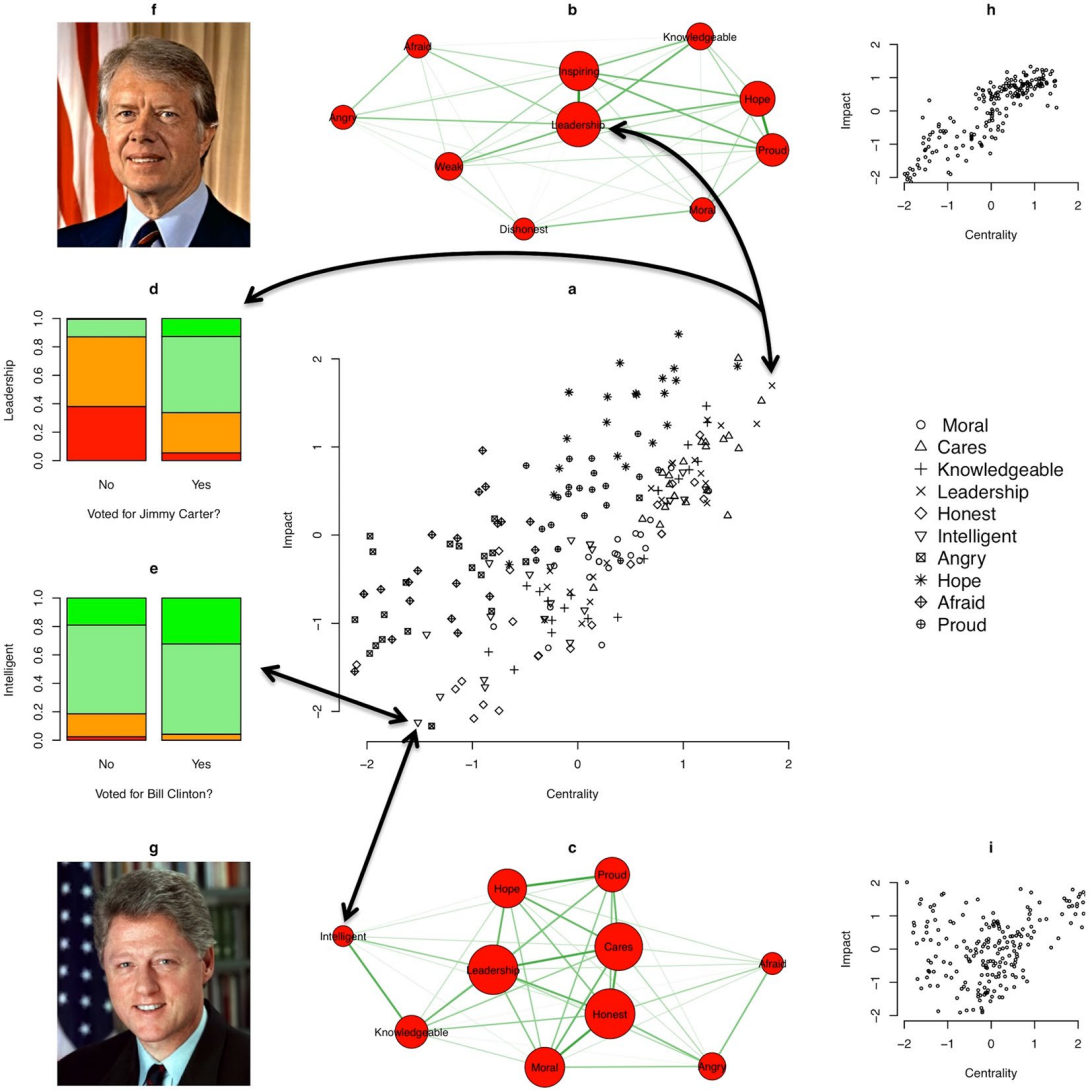
Central attitude elements have stronger impact on voting decisions than peripheral attitude elements. (**a**) Relation between centrality and impact of attitude elements. (**b**–**e**) Two illustrations of the analytic strategy to assess centrality and impact. (**b**,**c**) Attitude network toward Jimmy Carter (Bill Clinton) in 1980 (1992). The networks have the same characteristics as the networks shown in Fig. 2, except that the size of the nodes corresponds to the nodes’ relative centrality (the bigger the node, the higher its centrality). (**d**,**e**) Relation between endorsing the belief that Jimmy Carter (Bill Clinton) would provide strong leadership (is intelligent) and voting for Jimmy Carter (Bill Clinton). Colours of the bars represent the percentage of individuals who agree or do not agree with the judgment (the more green, the higher the agreement; the more red, the lower the agreement). See Table 1 for more information on the attitude elements. (**f**) Photo of Jimmy Carter by unknown photographer. Photo is under the CC0/Public Domain Licence. Source: https://www.goodfreephotos.com/people/jimmy-carter-portrait.jpg.php. (**g**) Photo of Bill Clinton by Bob McNeely. Photo is under the CC0/Public Doman Licence. Source: https://www.goodfreephotos.com/people/bill-clinton-portrait-photo.jpg.php. (**h**,**i**) Relation between centrality and impact for the simulated set of networks that was closest to the mean correlation plus (minus) one standard deviation.

Second, we tested whether central attitude elements (based on closeness) have more impact on behaviour. This impact was operationalized as the polychoric correlation between a given attitude element and the voting decision. We again standardized the centrality and impact estimates to exclude the possibility that results are driven by differences in mean centrality and mean impact. As can be seen in Fig. 3, we found a high positive correlation between standardized centrality and standardized impact (Pearson correlation: *r* = 0.70, *P* < 0.001), supporting our hypothesis.

### Forecast Analysis

**Figure 4:**
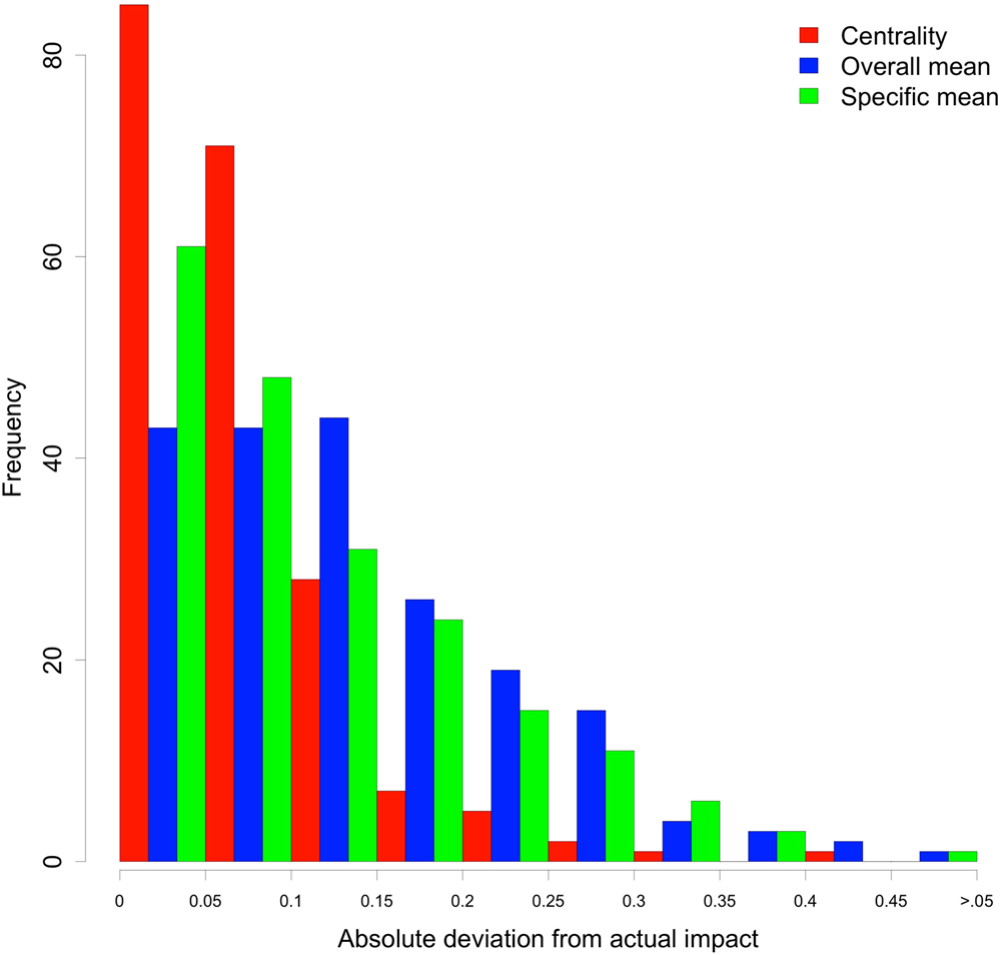
Accuracy of forecasts based on centrality, overall mean, and specific mean. The plot shows the results of forecasting the impact of each attitude element at each election.

To illustrate the practical relevance of our findings, we investigated whether centrality of attitude elements can be used to forecast the impact on voting decisions *before* knowing the outcome of the election (e.g., whether our analyses can be used to forecast the impact of attitude elements on the next presidential election). For each election (e.g., election of 2012), we estimated the regression parameters between impact and centrality from all elections except the forecasted election (e.g., 1980–2008). We calculated the predicted impact in the forecasted election using the centrality indices of the forecasted election and the regression parameters. As can be seen in Fig. 4, the predicted impact was very close to the actual impact (deviation median = 0.06, deviation interquartile range = 0.03–0.09) and outperformed both using the mean of all attitude elements (Deviation median = 0.12, deviation interquartile range = 0.06–0.18, Wilcoxon-matched pairs test: *V* = 3346, *P* < 0.001, CLES = 69.5%) and using the means of the specific attitude elements (Deviation median = 0.09, deviation interquartile range = 0.04–0.17, Wilcoxon-matched pairs test: *V* = 5057, *P* < 0.001, CLES = 65.2%). Using centrality thus creates the possibility to forecast the (almost) exact impact of an attitude element on the voting decision.

## Discussion

Starting in the 1930s with Richard T. LaPiere’s work^46^, attitude-behaviour consistency has been one of the central research themes in Social Psychology^3, 18, 19, 22, 41, 47^. While early work focused on the question *whether* attitudes drive or do not drive behaviour^47^, subsequent attitude researchers focused on *when* attitudes drive behaviour^19, 48–51^. This article provides a formalized and parsimonious answer to this question: The impact of attitudes on behaviour depends on the connectivity of the attitude network, with central attitude elements having the highest impact on behaviour within a given attitude network.

The present research has shown that network structure of attitudes can inform election campaign strategies (and behavioural change programs in general) by predicting both the extent to which individuals base their decision on their attitude and the extent to which an attitude element influences voting decision (and other behaviour relevant to an attitude). Connectivity can help inform how effective candidate-centred campaigns would be. High connectivity indicates that voting decisions highly depend on candidate attitudes, while low connectivity indicates that other factors may play a more substantial role than candidate attitudes (e.g., party identification^52, 53^, ideology^54, 55^, public policy issues^56–61^). Centrality can furthermore inform on the effectiveness of targeting specific attitude elements, as changing a central attitude element is probably more likely to affect the voting decision than changing a peripheral attitude element.

Future research might focus on how connectivity and centrality of attitude networks relate to other factors that influence voting decisions. Among the most important factors influencing voting decisions are party identification^52, 53^ and specific policy issues^56–61^. First, party identification might influence the connectivity of attitude networks, because it is likely that individuals, who identify with a political party, have a stronger drive for consistency in their attitudes toward presidential candidates. Party identification makes it also more likely that a given individual adopts a positive attitude toward the candidate of their party and it might also directly influence the voting decision. This makes party identification a possible confound of our results and we therefore also ran our analyses including only individuals, who do not identify with a political party. The results of this analysis mirrored the results in this paper (see Supplementary Note 3 and Figure 3). Second, policy issues might influence the centrality of attitude elements. If, for example, the current political climate is highly focused on foreign policies (e.g., the conflict in Syria), judging a candidate to be competent in respect to foreign policy making might take a central place in the attitude network. Generally, it is an important question for future research why some attitude elements are more central than others. Our analyses indicate that there are some attitude elements that are chronically central (see Fig. 3), with some variation that might be due to the specifics of the political climate during the different elections.

Another promising venture for future research would be to investigate how attitude networks develop during an election campaign. To do so, one could apply several intermediate assessments during the election campaign^62–64^. The use of such intermediate assessment was shown to improve the prediction of election outcomes^63^. How might attitude networks change during an election? Based on the CAN model, we expect that (a) the connectivity of attitude networks heighten during an election campaign and (b) attitude networks probably grow due to the addition of newly formed attitude elements^1^. Also, predictions regarding the success of an election campaign to change a given person’s attitude can be derived from the CAN model. Individuals holding attitudes that are based on highly connected networks already at the beginning of an election campaign are likely to not change their attitudes. Election campaigns might thus benefit from focusing on individuals holding attitudes that are based on weakly connected networks^65^.

In a broader sense, the CAN model advances our understanding of the relation between attitudes and behavioural decisions. Because the CAN model is a general model of attitudes, the results reported here likely generalize to other attitudes and behavioural decisions than those studied here as well. Using connectivity of attitude networks and centrality of attitude elements may for example provide more insight into issues such as which factors drive individuals to continue or stop smoking, buy a certain product, or behave aggressively toward a minority group. Furthermore, connectivity of attitude networks might unify the different approaches to explain variations in attitude-behaviour consistency, as it is likely that network connectivity is the glue that holds these factors together^1^ and because our results indicate that network connectivity comprehensively explains variations in attitude-behaviour consistency. Several predictions above and beyond the findings reported here can also be derived from the network structure of attitudes. For example, network structure predicts when and which persuasion attempts will be succesful^1^. Network theory thus holds great promise for advancing our understanding of the dynamical and structural properties of attitudes and their relation to a plethora of consequential human behaviours.

## Methods

### Simulation of networks

The simulation of networks followed four steps. First, an unweighted ‘base’ network consisting of 11 variables was created based on preferential attachment^7, 43^, the Small-World network model^8^, or the Erdos-Rényi random graph model^44^ using the R package iGraph^66^. The preferential attachment algorithm starts with one node and then adds one node in each time step. The probability to which nodes the new node connects depends on the degree of the old node:

3 $$Pr(i)=\frac{k{(i)}^{\alpha }+1}{\sum _{j}k{(j)}^{\alpha }+1},$$

where *k*(*i*) is the degree of a given node. *α* was set to vary uniformly between 0.30 and 0.70. At each time step *m* edges were added to the network. *m* was set to vary uniformly between 4 and 6 (resulting in relatively dense networks, as was shown to be the case for attitude networks^1^). The Small-World network model starts with a ring lattice with nodes being connected to *n* neighbours and then randomly rewires edges with a *p* probability. *n* was set to uniformly vary between 3 and 4 and *p* was set to uniformly vary between 0.05 and 0.10. In the Erdos-Rényi graph, nodes are randomly connected by a given number of edges. Number of edges was set to uniformly vary between 30 and 45.

Second, edge weights were added to the base network. To have psychometrically realistic edge weights, we drew edge weights from either a normal distribution with *M* = 0.15 and *SD* = 0.0075, a Pareto power law distribution with α = 3 and β = 0.10, or a uniform distribution with range of 0.01–0.30.

Third, we created 20 variations of the weighted base network, in which the temperature of the Ising model was varied. The inverse temperature parameter *β* was drawn from a normal distribution with *M* = 1 and *SD* = 0.2 (with higher numbers representing low entropy). To ensure that all nodes have roughly the same variance, we drew thresholds of nodes from a normal distribution with *M* = 0 and *SD* = 0.25.

Fourth, using the R-package IsingSampler^16^, 1000 individuals for each of the variations of the base network were simulated based on the probability distribution implied by the Ising model. This procedure was repeated 100 times and each set of 20 variations of the different 100 base networks was analysed separately.

### Participants

The open-access data of the ANES involves large national random probability samples. Data were each collected in two interviews – one before and one after each presidential election from 1980 to 2012 – by the Center for Political Studies of the University of Michigan. In total, 21,365 participants participated in these nine studies (for *N*s per study see Supplementary Table 2), of which 16,667 participants stated that they voted for president. Non-voters were excluded from the analyses, because we assume that the decision whom to vote for is more likely to be part of the attitude network than the decision whether to vote or not. In Supplementary Note 2, however, we show that similar findings are obtained when non-voters are included in the analysis.

### Measures

In each of the studies between six and 16 items tapping beliefs and between four and eight items tapping feelings toward the presidential candidates were assessed in the pre-election interviews. Feelings were assessed on two-point scales and beliefs were assessed on four-point scales (in a subsample of the ANES of 2008 and in the ANES of 2012, beliefs were assessed on a five-point scale). To have comparable attitude networks between the different elections, we always used six items tapping beliefs and four items tapping feelings (see Table 1 for a list of included attitude elements). In the post-election interview, participants were asked which candidate they voted for. Depending on which presidential candidate the analysis focused, we scored the response as 1 when the participant stated that they voted for the given candidate and we scored the response as 0 when the participant did not vote for the given candidate.

### Statistical Analyses

We performed the same statistical analyses on the simulated and empirical data.

#### Network estimation

Attitude networks were estimated using zero-order polychoric (tetrachoric) correlations between the (simulated) attitude elements as edge weights. We chose to use zero-order correlations as edge weights instead of estimating direct causal paths between the attitude elements because our simulations have shown that attitude networks based on zero-order correlations perform better than techniques that provide an estimate of the underlying causal network^67^.

#### Network descriptives

Both the ASPL and closeness are based on shortest path between lengths (*d*) between nodes. To calculate shortest path lengths, we used Dijkstra’s algorithm^68^, implemented in the R package qgraph^69^:

4 $${d}^{w}(i,j)=\,\min (\frac{1}{{w}_{ih}}+\frac{1}{{w}_{hj}}).$$

ASPL is then the average of the shortest path lengths between each pair of nodes in the network. Closeness (*c*) was calculated using the algorithm for weighted networks developed by Opsahl, Agneessens, and Skvoretz^35^, using the R package qgraph:

5 $$c(i)={[\sum _{j}^{N}d(i,j)]}^{-1}.$$

#### Impact estimates

To estimate average impact of (simulated) attitude elements on (simulated) voting decisions, we calculated the biserial correlation between the sum score of (simulated) attitude elements and the (simulated) voting decision. We then calculated the Pearson correlation between connectivity and average impact for the 20 networks in the empirical study and for each set of 20 variations of the base networks in the simulation study. The clearly linear relation between connectivity and impact justified the use of Pearson correlation and significance testing. For the simulation study, we calculated the mean and standard deviation of the correlations obtained for each set of variations of the base network.

To estimate the impact of a given (simulated) attitude element on the (simulated) voting decision, we calculated the zero-order polychoric correlation between a given (simulated) attitude element and the (simulated) voting decision. We then calculated the Pearson correlation between standardized centrality and standardized impact of the attitude elements in the empirical study and for each set of 20 variations of the base networks in the simulation study. The clearly linear relation between centrality and impact justified the use of Pearson correlation and significance testing. For the simulation study, we calculated the mean and standard deviation of the correlations obtained for each set of variations of the base network.

#### Forecast analysis

For the forecast analysis, we first conducted nine regression analyses, in which impact was regressed on centrality. In each regression analysis, the forecasted election was omitted. From each of the regression equations, we first extracted the beta and intercept coefficients. Second, we multiplied the centrality indices of the forecasted election with the beta coefficient and added the intercept coefficient. Note that not in every election the same attitude elements were assessed. Of the ten used attitude elements, seven were assessed at each election. For the remaining three, we grouped the attitude elements together that were most similar to each other (see Table 1). Third, we compared the resulting estimates with the actual impact of the attitude elements and calculated the absolute deviance scores. We then compared the performance of the centrality prediction to the overall mean prediction and the specific mean prediction. For both these predictions, we again calculated predictions nine times, omitting one of the elections each time. For the overall mean prediction, we calculated the mean of all attitude elements and for the specific mean prediction, we calculated the mean of each specific attitude element. We tested whether the centrality prediction performed better than the overall mean prediction and the specific mean prediction using Wilcoxon signed-rank tests.

Missing values were deleted casewise (Supplementary Table 2 shows the number of excluded participants per attitude network). Most missing values stemmed either from participants responding to an item that they did not know the answer or from non-participation during the post-election interview. Few missing values stemmed from interview errors.

We also ran several alternative analyses that confirmed the robustness of our results: We ran alternative analyses on non-voters (see Supplementary Note 2 and Figure 2), on independents (see Supplementary Note 3 and Figure 3), on missing values (see Supplementary Note 4 and Figure 4), on networks with different number of nodes (see Supplementary Note 5 and Figure 5), and on latent variable models (see Supplementary Note 6 and Table 3).

## Electronic supplementary material

Supplementary Information
