# Supplementary material for: Network Structure Explains the Impact of Attitudes on Voting Decisions
Source: Sci Rep. 2017 Jul 7;7:4909. doi: 10.1038/s41598-017-05048-y (PMC5501836; doi:10.1038/s41598-017-05048-y)
Supplement: Supplementary file 1 — Supplementary Information [file 41598_2017_5048_MOESM1_ESM.pdf]

Supplementary Information: Network Structure Explains the Impact of Attitudes on Voting Decisions

Jonas Dalege, Denny Borsboom, Frenk van Harreveld, Lourens J. Waldorp & Han L. J. van der Maas

Department of Psychology, University of Amsterdam, 1018 WT Amsterdam, The Netherlands

Supplementary Figures

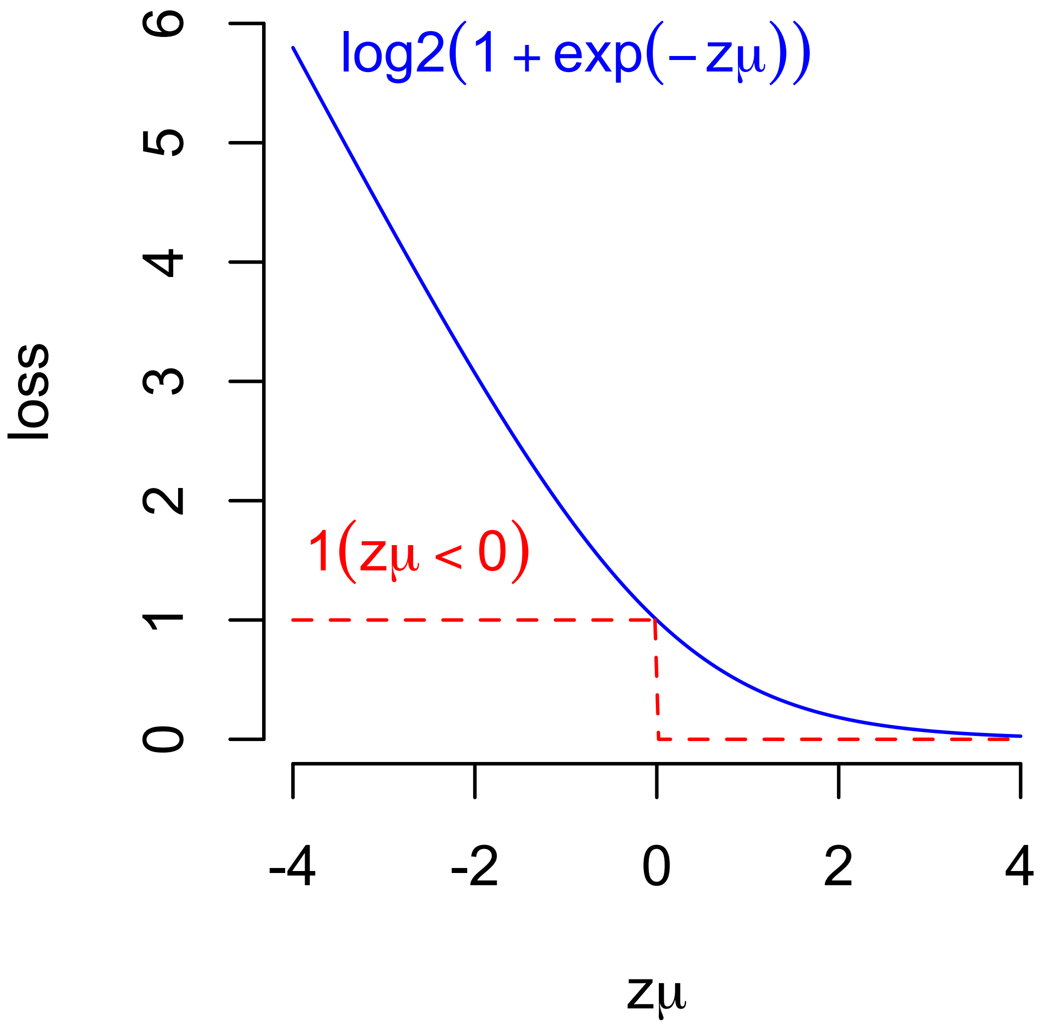

Supplementary Figure 1: Misclassification loss $\mathcal{C}$ in (7) and ψ in (6) as a function of the margin $x\mu = (2y - 1)\mu$.

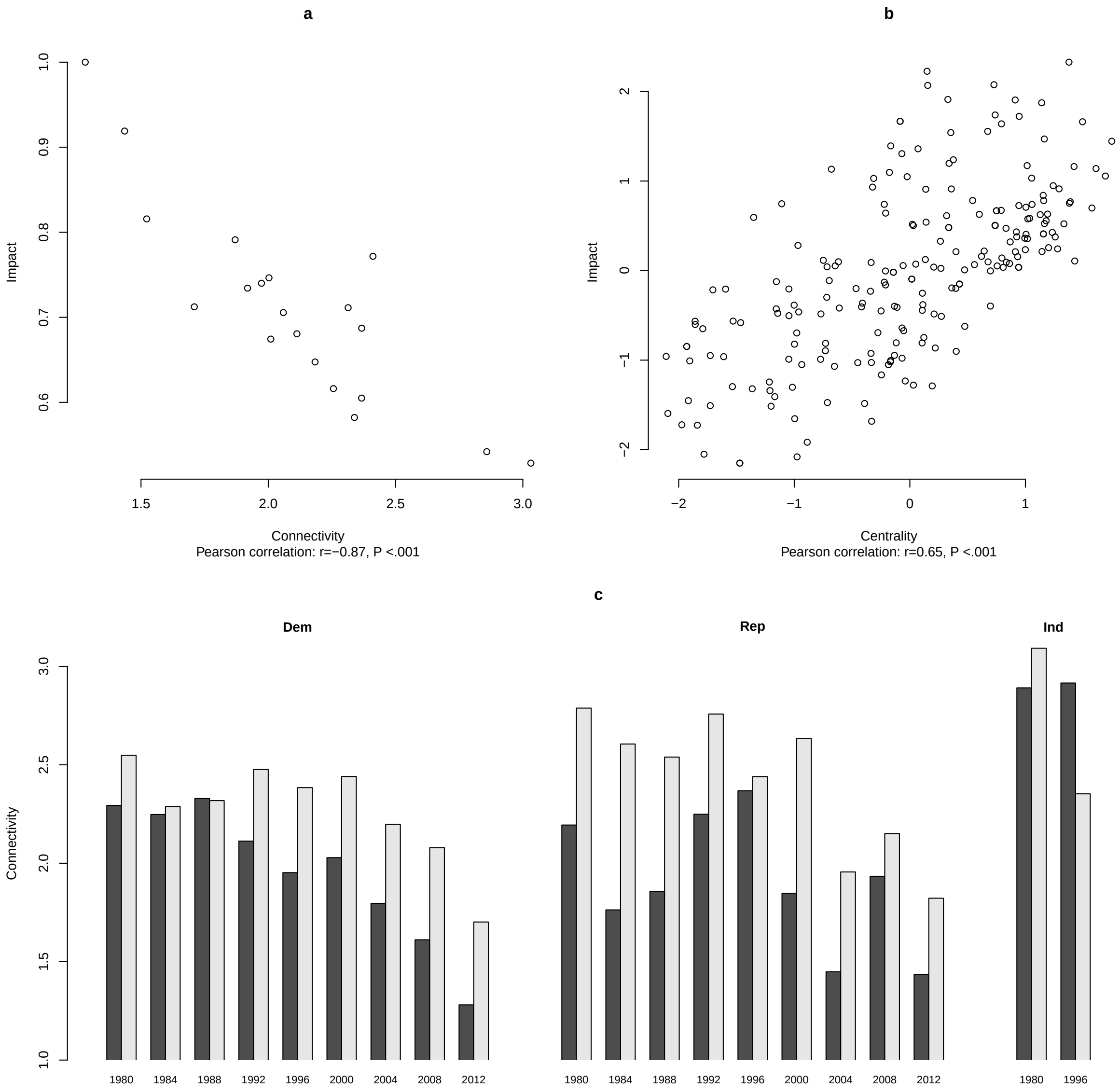

Supplementary Figure 2: Analyses including non-voters. (**a**) Relation between connectivity and global impact. (**b**) Relation between centrality and specific impact. (**c**) Comparison between connectivity of voters (dark grey bars) and non-voters (light grey bars). Dem: Democratic candidates; Rep: Republican candidates, Ind: Independent candidates.

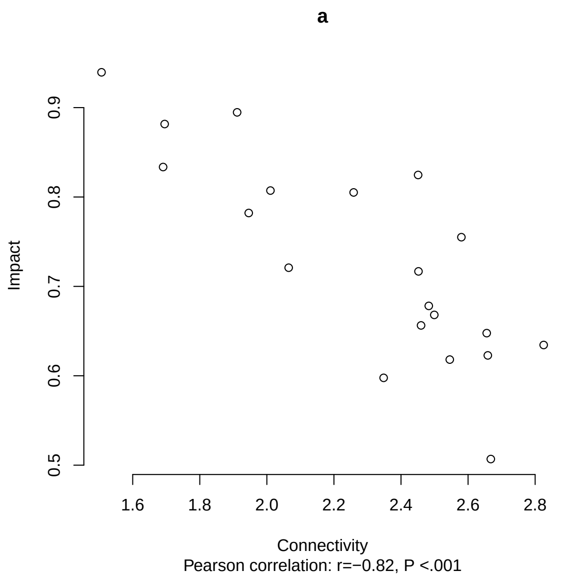

Supplementary Figure 3: Analyses including only independents (**a**) Relation between connectivity and global impact. (**b**) Relation between centrality and specific impact.

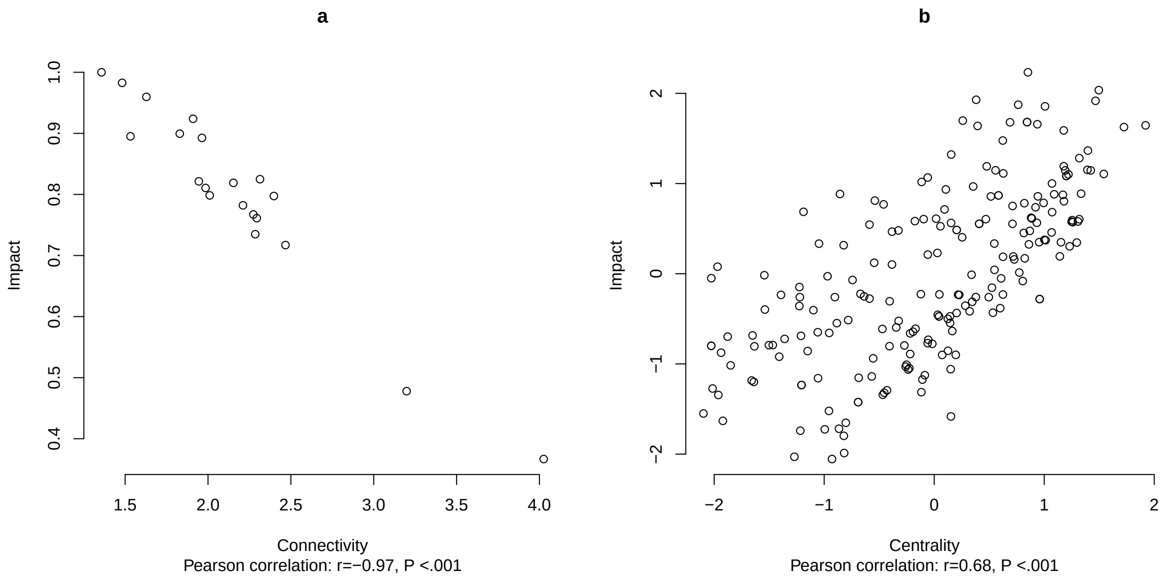

Supplementary Figure 4: Analyses with imputed missing values (**a**) Relation between connectivity and global impact. (**b**) Relation between centrality and specific impact.

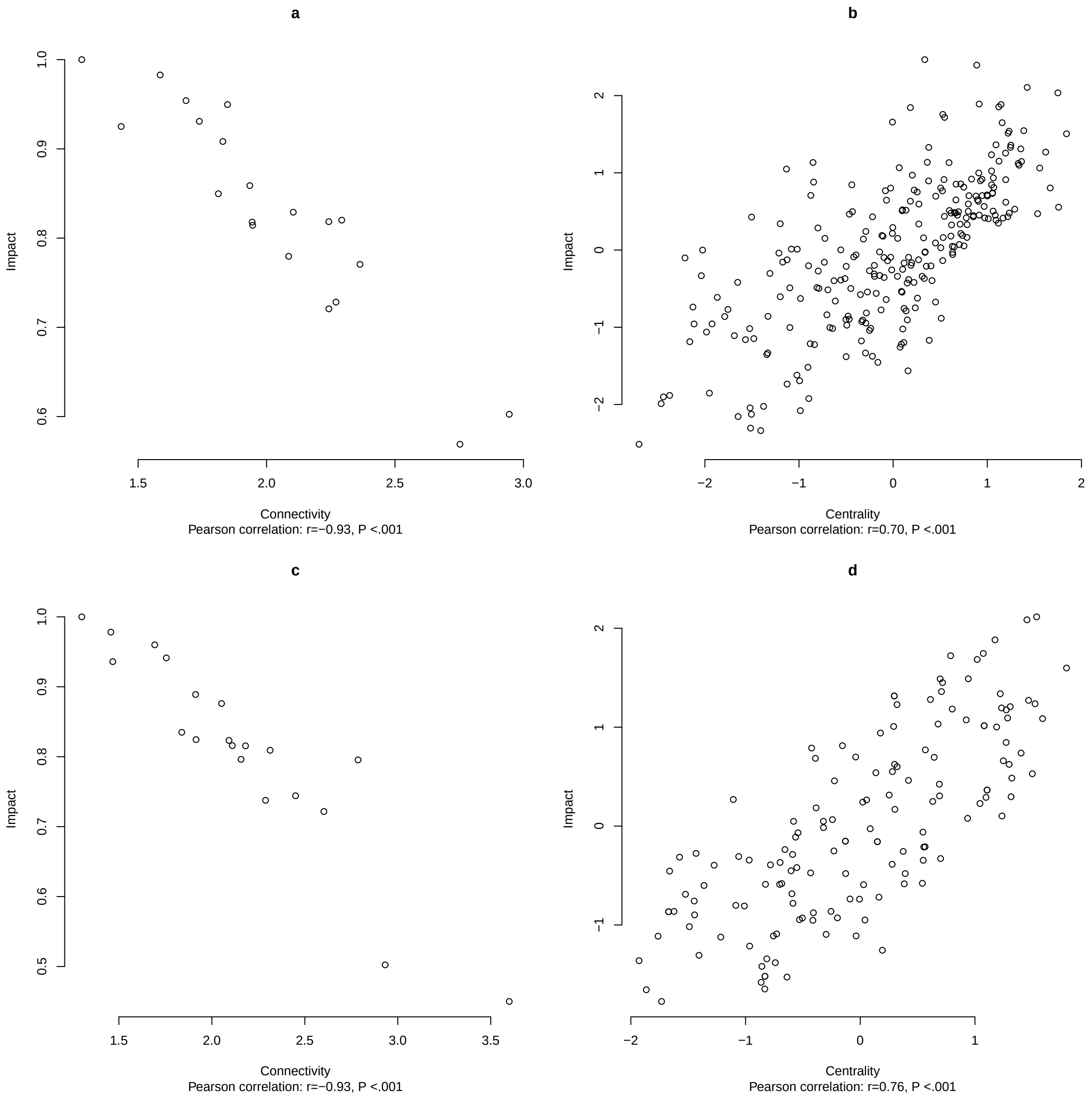

Supplementary Figure 5: Analyses on different numbers of attitude elements (**a**) Relation between connectivity and global impact for the analysis including all attitude elements. (**b**) Relation between centrality and specific impact for the analysis including all attitude elements. (**c**) Relation between connectivity and global impact for the analysis including seven attitude elements. (**d**) Relation between centrality and specific impact for the analysis including seven attitude elements.

Supplementary Tables

Supplementary Table 1: Results of the Simulations for Each Network-Generating Algorithm and Edge Weight Distribution.

	Preferential attachment	Small-world	Random graph
	Connectivity/impact correlations		
Normal distribution	mean r=-0.91	mean r=-0.91	mean r=-0.90
	s.d. r=0.07	s.d. r=0.05	s.d. r=0.08
Power-law distribution	mean r=-0.92	mean r=-0.91	mean r=-0.91
	s.d. r=0.05	s.d. r=0.05	s.d. r=0.04
Uniform distribution	mean r=-0.92	mean r=-0.89	mean r=-0.90
	s.d. r=0.05	s.d. r=0.08	s.d. r=0.08
	Centrality/impact correlations		
Normal distribution	mean r=0.72	mean r=0.51	mean r=0.57
	s.d. r=0.18	s.d. r=0.33	s.d. r=0.27
Power-law distribution	mean r=0.70	mean r=0.46	mean r=0.60
	s.d. r=0.19	s.d. r=0.34	s.d. r=0.23
Uniform distribution	mean r=0.68	mean r=0.49	mean r=0.60
	s.d. r=0.24	s.d. r=0.29	s.d. r=0.25

Supplementary Table 2: Number of participants per election and number of participants with missing values.

Election	N complete sample	N non-voters	N with missing values (Democratic candidates)	N with missing values (Republican candidates)
1980	1,614	411	338	396
1984	2,257	539	583	435
1988	2,040	545	470	477
1992	2,485	562	567	358
1996	1,714*	374	239	318
2000	1,807	376	440	493
2004	1,212	231	282	195
2008	2,322	509	368	390
2012	5,914	1,141	557	644

Note. *Of these 1,714 participants, 1,316 participants also participated during the election of 1992.

Supplementary Table 3: Fit measures of the latent variable models.

Candidate	One-factor model	Hierarchical model
Carter 1980	$\chi(35) = 1030.53$, $CFI = 0.80$, $RMSEA = 0.18$	$\chi(30) = 528.58$, $CFI = 0.90$, $RMSEA = 0.14$
Reagan 1980	$\chi(35) = 411.40$, $CFI = 0.91$, $RMSEA = 0.12$	$\chi(30) = 198.74$, $CFI = 0.96$, $RMSEA = 0.08$*
Anderson 1980	$\chi(35) = 655.19$, $CFI = 0.75$, $RMSEA = 0.18$	$\chi(30) = 290.38$, $CFI = 0.90$, $RMSEA = 0.12$*
Mondale 1984	$\chi(35) = 1410.69$, $CFI = 0.79$, $RMSEA = 0.19$	$\chi(31) = 859.75$, $CFI = 0.87$, $RMSEA = 0.15$
Reagan 1984	$\chi(35) = 1730.83$, $CFI = 0.83$, $RMSEA = 0.19$	$\chi(31) = 1194.73$, $CFI = 0.88$, $RMSEA = 0.17$*
Dukakis 1988	$\chi(35) = 1166.68$, $CFI = 0.81$, $RMSEA = 0.18$	$\chi(31) = 683.11$, $CFI = 0.89$, $RMSEA = 0.14$
Bush 1988	$\chi(35) = 940.98$, $CFI = 0.87$, $RMSEA = 0.16$	$\chi(31) = 593.40$, $CFI = 0.92$, $RMSEA = 0.13$*
Clinton 1992	$\chi(35) = 1536.91$, $CFI = 0.83$, $RMSEA = 0.18$	$\chi(31) = 995.36$, $CFI = 0.89$, $RMSEA = 0.15$*
Bush 1992	$\chi(35) = 1304.31$, $CFI = 0.85$, $RMSEA = 0.15$	$\chi(31) = 840.63$, $CFI = 0.91$, $RMSEA = 0.13$*
Clinton 1996	$\chi(35) = 1492.00$, $CFI = 0.83$, $RMSEA = 0.19$	$\chi(31) = 908.92$, $CFI = 0.90$, $RMSEA = 0.16$*
Dole 1996	$\chi(35) = 1312.91$, $CFI = 0.80$, $RMSEA = 0.19$	$\chi(31) = 842.52$, $CFI = 0.87$, $RMSEA = 0.16$
Perot 1996	$\chi(35) = 428.94$, $CFI = 0.78$, $RMSEA = 0.18$	Fit measures could not be computed*
Gore 2000	$\chi(35) = 1401.65$, $CFI = 0.79$, $RMSEA = 0.19$	$\chi(31) = 837.05$, $CFI = 0.88$, $RMSEA = 0.16$
Bush 2000	$\chi(35) = 1080.12$, $CFI = 0.84$, $RMSEA = 0.18$	$\chi(31) = 685.86$, $CFI = 0.90$, $RMSEA = 0.15$
Kerry 2004	$\chi(35) = 771.78$, $CFI = 0.86$, $RMSEA = 0.17$	$\chi(31) = 458.36$, $CFI = 0.92$, $RMSEA = 0.14$*
Bush 2004	$\chi(35) = 1100.80$, $CFI = 0.87$, $RMSEA = 0.20$	$\chi(31) = 661.61$, $CFI = 0.92$, $RMSEA = 0.16$*
Obama 2008	$\chi(35) = 1373.76$, $CFI = 0.90$, $RMSEA = 0.16$	$\chi(31) = 642.16$, $CFI = 0.95$, $RMSEA = 0.12$*
McCain 2008	$\chi(35) = 1553.26$, $CFI = 0.86$, $RMSEA = 0.18$	$\chi(31) = 863.92$, $CFI = 0.92$, $RMSEA = 0.14$
Obama 2012	$\chi(35) = 5863.21$, $CFI = 0.91$, $RMSEA = 0.20$	$\chi(31) = 2428.10$, $CFI = 0.96$, $RMSEA = 0.14$
Romney 2012	$\chi(35) = 5781.98$, $CFI = 0.88$, $RMSEA = 0.20$	$\chi(31) = 3021.35$, $CFI = 0.94$, $RMSEA = 0.15$

Note. *The covariance matrices of the latent variables of these models were not positive definitive. This indicates poor fit to the data.

Supplementary Notes

Supplementary Note 1: Analytical Solutions of the Hypotheses

In the Ising model the intuition is that higher connection strength will allow for better prediction. Here we show this intuition is correct.

Logistic regression and the Ising model. The Ising model is part of the exponential family of distributions[1–3]. Let G be a graph consisting of nodes in $V = \{1, 2, \ldots, p\}$ and edges (s,t) in $E \subseteq V \times V$. To each node $s \in V$ a random variable X_s is associated with values in $\{0,1\}$. The probability of each configuration x depends on a main effect (external field) and pairwise interactions. It is sometimes referred to as the auto logistic-function[4], or a pairwise Markov random field, to emphasise that the parameter and sufficient statistic space are limited to pairwise interactions[3]. Each $x_s \in \{0,1\}$ has conditional on all remaining variables (nodes) $X_{\setminus s}$ probability of success $\pi_s := \mathbb{P}(X_s = 1 \mid x_{\setminus s})$. The distribution for configuration x of the Ising model is then

$$\mathbb{P}(x) = \frac{1}{Z(\theta)} \exp\left(\sum_{s \in V} m_s x_s + \sum_{(s,t)\in E} A_{st} x_s x_t \right) \tag{1}$$

which is clearly of the form of exponential family. In general, the normalisation $Z(\theta)$ is intractable, because the sum consists of 2^p possible configurations for $y \in \{0,1\}^p$; for example, for $p = 30$ we obtain over 1 million configurations to evaluate in the sum in $Z(\theta)$ (see[3] for lattice [Bethe] approximations).

The conditional distribution is again an Ising model[4,5]

$$\pi_s = \mathbb{P}(x_s = 1 \mid x_{\setminus s}) = \frac{\exp\left(m_s + \sum_{t:(s,t)\in E} A_{st} x_t\right)}{1 + \exp\left(m_s + \sum_{t:(s,t)\in E} A_{st} x_t\right)}. \tag{2}$$

It immediately follows that the log-odds[4] is

$$\mu_s(x_{\setminus s}) = \log\left(\frac{\pi_s}{1 - \pi_s}\right) = m_s + \sum_{t:(s,t)\in E} A_{st} x_t. \tag{3}$$

Note that the log-odds $\theta \mapsto \mu_\theta$ is a linear function, and so if $x = (1, x_{\setminus s})$ then $\mu_\theta = x^\mathsf{T}\theta$.

Recall that $\theta \mapsto \mu_\theta$ is the linear function $\mu_{\theta_s}(x_{\setminus s}) = m_s + \sum_{t \in V \setminus s} A_{st} x_t$ of the conditional Ising model obtained from the log-odds (3). Define $\mu_s := \mu_{\theta_s}(x_{\setminus s})$. We use the notation that the node of interest $x_{i,s}$ is denoted by y_i and we let the remaining variables and a 1 for the intercept be indicated by $x_i = (1, x_{i,\setminus s})$, basically leaving out the subscript s to index the node, and only use it whenever circumstances demand it. Let the loss function be the negative log of the conditional probability π in (2), known as a pseudo log-likelihood[4]

$$\psi(x,\mu) := -\log \mathbb{P}(y \mid x) = -x\mu + \log(1 + \exp(\mu)). \tag{4}$$

Monotonicity of prediction loss as a function of connectivity. In logistic regression there is a natural classifier that predicts whether y_i is 1 or 0. We simply check whether

the probability of a 1 is greater than 1/2, that is, whether $\pi_i > 1/2$. Because $\mu_i > 0$ if and only if $\pi_i > 1/2$ we obtain the natural classifier

$$\mathcal{C}(y_i) = \mathbb{1}\{\mu_i > 0\} \tag{5}$$

where $\mathbb{1}$ is the indicator function. This is 0-1 loss[6]. Sometimes the margin interpretation is used where the log of the conditional probability $\pi_{i,s}$ is used with variables in $\{-1,1\}$ (see[7]) . Let $z = 2y - 1$ such that for $x \in \{0,1\}$ we obtain $z \in \{-1,1\}$. The loss ψ (pseudo log-likelihood) in (4) can then be rewritten as

$$\psi(z_i, \mu_i) = \log(1 + \exp(-z_i \mu_i)). \tag{6}$$

Often the logarithm with base 2 is chosen since then $\psi(z_i, 0) = 1$. The classification translates to

$$\mathcal{C}(z_i) = \mathbb{1}\{z_i \mu_i > 0\}. \tag{7}$$

Logistic loss ψ in (6) is an upper bound to $\mathcal{C}$ in (7), and is 1 at the value of the margin $z_i \mu_i = 0$, as shown in Supplementary Figure 1. Here we use logistic loss ψ as defined in (4) because it is more common. This function is strictly monotone decreasing.

It follows immediately from monotonicity that $\psi(z_i, \mu) > \psi(z_i, \mu^*)$ if $\mu < \mu^*$. Of course, we have the same for the 0-1 loss: $\mathbb{1}\{z_i \mu_i > 0\} \geq \mathbb{1}\{z_i \mu_i^* > 0\}$ if $\mu < \mu^*$.

If the average degree of each node were subtracted from the Hamiltonian μ_s, then we obtain the Ising model without an external field. If we have

$$m_s = -\frac{1}{2} \sum_{t:(s,t) \in E} A_{st}$$

then we see that

$$\mu_s = -\frac{1}{2} \sum_{t:(s,t) \in E} A_{st} + \sum_{t:(s,t) \in E} A_{st} x_s x_t = \sum_{t:(s,t) \in E} A_{st}(x_s x_t - 1/2)$$

Switching to the labeling $z = 2y - 1 \in \{-1,1\}$, we obtain that the average is 0 (implying $m_s = 0$), and so the Hamiltonian consists only of the interactions $\sum_{t:(s,t) \in E} A_{st} z_s z_t$. And so if $A_{st} \geq 0$ for all $s, t \in V$ then $\mu_s^* > \mu_s$ iff $A_{st}^* > A_{st}$, i.e., the Hamiltonian is larger if and only of the connectivity is larger. As seen above, this leads immediately to the monotonicity above.

Closeness and correlations Let $d_{st}(r) = \min\{1/r_{si} + 1/r_{ij} + \cdots + 1/r_{kt} : \forall i, j, k \in V \backslash \{s, t\}\}$ be the shortest distance in terms of Dijkstra's algorithm[8], where r_{ij} is the weight, in our case a (polychromic) correlation that are all positive. Then closeness is defined as

$$c_s(r) = \left(\sum_{t \in V \backslash \{s\}} d_{st}(r) \right)^{-1}$$

The intuition is here that a node with high closeness will have connections or paths to other nodes with high correlations (weights). To see the intuition, consider node s being

connected only to node t with correlation r_{st}. Then $c_s(r) = r_{st}$; if this correlation is high, then so is the closeness of this node. If there is more than one connection, we see that the shortest path $d_{st}(r)$ is low if all correlations r_{ij} are high (close to 1), implying that closeness $c_s(r)$ is high.

Suppose we have two sets weights R_1 and R_2, inducing two graphs G_1 and G_2 with the same nodes and edge sets but with different weights. We pick a path between nodes s and t, denoted by $P_{st} = \{(x_0 = s, x_1), (x_1, x_2), \dots, (x_{k-1}, x_k = t)\}$ of length k. Suppose that for this path we have

$$\sum_{i=0}^{k} r_{1,i-1,i} \geq \sum_{i=0}^{k} r_{2,i-1,i}$$

Then it follows that

$$\sum_{i=0}^{k} \frac{1}{r_{1,i-1,i}} \leq \sum_{i=0}^{k} \frac{1}{r_{2,i-1,i}}$$

In other words, the higher the correlations the higher the closeness. This does not imply that any randomly drawn node connected to a node with high closeness will have a high correlation, only that on average the correlations will be higher if they are connected to a node with high closeness than if they are connected to a node with low closeness.

Supplementary Note 2: Alternative Analysis on Non-Voters

To investigate whether our results are robust to the inclusion of non-voters, we performed the same analyses but now including non-voters and labelling them as voters *against* the focal candidates. The results of this analysis mirrored the results reported in the Results section: The correlation between connectivity and average impact remained high and significant (see Supplementary Figure 1a). The same holds for the correlation between centrality and impact (see Supplementary Figure 1b). The predicted impact remained very close to the actual impact (deviation median=0.06, deviation interquartile range=0.02-0.09) and outperformed both using the mean of all attitude elements (deviation median=0.10, deviation interquartile range=0.05-0.18, Wilcoxon-matched pairs test: V=4006, P<0.001, CLES=69.5%) and using the means of the specific attitude elements (deviation median=0.08, deviation interquartile range=0.04-0.15, Wilcoxon-matched pairs test: V=5670, P<0.001, CLES=64.7%).

We also tested another prediction from our model regarding differences between voters and non-voters: That voters are expected to have a more densely connected network than non-voters. As can be seen in Supplementary Figure 1c, attitude networks of voters were much more highly connected (mean=2.03, s.d.=0.43) than attitude networks of non-voters (mean=2.38, s.d.=0.34, Student's t-test: T=2.86, P<0.001, Cohen's D=0.91).

Supplementary Note 3: Alternative Analysis on Independents

As our analysis are correlational, it is important to exclude the possibility that third variables affected the relations tested in this paper. The most likely variable to be such a confound is party identification. It is, for example, easy to imagine that party identification might affect the connectivity of attitude networks, the valence of the attitude, and for whom a person votes. We therefore reran our analyses including only participants, who do not identify with any political party. The results of this analysis mirrored the results reported in the Results section: The correlation between connectivity and average impact remained high and significant (see Supplementary Figure 2a). The same holds for the correlation between centrality and impact (see Supplementary Figure 2b). The predicted impact remained very close to the actual impact (deviation median=0.06, deviation interquartile range=0.03-0.11) and outperformed both using the mean of all attitude elements (deviation median=0.11, deviation interquartile range=0.05-0.17, Wilcoxon-matched pairs test: V=4152, P<0.001, CLES=66.2%) and using the means of the specific attitude elements (deviation median=0.09, deviation interquartile range=0.04-0.16), Wilcoxon-matched pairs test: V=5703, P<0.001, CLES=62.7%).

Supplementary Note 4: Alternative Analysis on Missing Values

To investigate whether our results are robust to imputation of missing values, we reran our analyses with imputing missing values using Predictive Mean Matching[9,10]. The results of this analysis mirrored the results reported in the Results section: The correlation between connectivity and average impact remained high and significant (see Supplementary Figure 3a). The same holds for the correlation between centrality and impact (see Supplementary Figure 3b). The predicted impact remained very close to the actual impact (deviation median=0.07, deviation interquartile range=0.03-0.11) and outperformed both using the mean of all attitude elements (deviation median=0.12, deviation interquartile range=0.05-0.19, Wilcoxon-matched pairs test: V=3826, P<0.001, CLES=68.8%) and using the means of the specific attitude elements (deviation median=0.09, deviation interquartile range=0.04-0.18, Wilcoxon-matched pairs test: V=5526, P<0.001, CLES=64.9%).

Supplementary Note 5: Alternative Analysis on Networks Based on Different Numbers of Attitude Elements

To investigate whether our results are robust to our choice of attitude elements, we reran our analyses based on all available attitude elements (note that in this case the forecast analyses are not possible because for these analyses the same number of attitude elements for each election is necessary) and on the seven attitude elements that were assessed at each election. The results of these analyses mirrored the results reported in the Results section: For the analysis including all attitude elements, the correlation between connectivity and average impact remained high and significant (see Supplementary Figure 4a). The same holds for the correlation between centrality and impact (see Supplementary Figure 4b).

For the analysis including the seven attitude elements that were assessed at each election, the correlation between connectivity and average impact remained high and significant (see Supplementary Figure 4c). The same holds for the correlation between centrality and impact (see Supplementary Figure 4d). The predicted impact remained very close to the actual impact (deviation median=0.06, deviation interquartile range=0.03-0.09) and outperformed both using the mean of all attitude elements (deviation median=0.11, deviation interquartile range=0.06-0.18, Wilcoxon-matched pairs test: V=1505, P<0.001, CLES=68.7%) and using the means of the specific attitude elements (deviation median=0.09, deviation interquartile range=0.05-0.16), Wilcoxon-matched pairs test: V=2341, P<0.001, CLES=65.2%).

Supplementary Note 6: Alternative Analysis on Latent Variable Models

One might argue that the results reported in this article can also be expected when attitudes are conceptualized as latent variables and the responses on attitude elements are treated as indicators of the latent attitude. From this perspective, high (low) centrality of attitude elements would indicate high (low) factor loadings on the latent variable attitude and high (low) connectivity would indicate high (low) average factor loadings. In a purely statistical sense, this objection would be correct as factor loadings also reflect how much information a given attitude element holds on all other attitude elements. However, in our view the latent variable framework does not provide a sensible alternative for a data-generating model of the hypotheses put forward here. For such a model, one would have to assume that the latent variable attitude acts as common cause of the attitude elements. This assumption, however, is at odds with several key concepts in the attitude literature[11], such as cognitive consistency[12], ambivalence[13], and the idea that attitudes are formed by attitude elements[14,15].

To further rule out that the latent variable framework provides an alternative explanation of our results, we investigated the fit of latent variable models on the data reported in this article. For each attitude toward each candidate at each election, we fitted two latent variable models. First, we fitted a one-factor model with all attitude elements loading on this single factor representing a latent attitude. Second, we fitted a hierarchical factor model with three or four first-order factors and one second-order factor representing a latent attitude. We fitted the hierarchical factor model because earlier research indicated that beliefs and feelings form different factors[16] and that negative and positive attitude elements form different factors[17]. In most of the data sets used here, no negative beliefs were assessed. For these data sets, we fitted a hierarchical factor model with beliefs, negative feelings, and positive feelings loading on different first-order factors, respectively. For the data sets in which negative beliefs were assessed, we fitted a hierarchical factor model with negative beliefs, positive beliefs, negative feelings, and positive feelings loading on different first-order factors, respectively. As can be seen in Supplementary Table 3, both the one-factor models and the hierarchical models fitted poorly. The latent variable framework thus appears to be an unlikely alternative explanation of our results.

This discussion on whether our results can also be explained by the latent variable framework is somewhat reminiscent of the discussion regarding the idea that instability of attitudinal responses is indicative of individuals holding nonattitudes[18]. Several critiques of this idea pointed out that when measurement error is accounted for, individuals, who seemingly hold nonattitudes, show stable attitudes[19–21]. A similar critique might apply to our findings. It is our view, however, that two findings speak against this critique. First, if we assume that the intercorrelations of attitude elements are determined only (or foremost) by measurement error, then the factor models we fitted should show good fit. This was clearly not the case. Second, systematic variation of intercorrelations would not be expected from the measurement error perspective. Thus, our finding that connectivity of attitudes correlates almost perfectly with the attitude's impact on behaviour would not be expected from the measurement error perspective.

References

1. Brown, L. *Fundamentals of Statistical Exponential Families.* (Institute of Mathematical Statistics, Hayward, 1986).

2. Young, G. & Smith, R. *Essentials of Statistical Inference.* (Cambridge Univ. Press, 2005).

3. Wainwright, M. J. & Jordan, M. I. Graphical models, exponential families, and variational inference. *Found. Trends®Mach. Learn.* **1,** 1-305 (2008).

4. Besag, J. Spatial interaction and the statistical analysis of lattice systems. *J. R. Stat. Soc. Ser. B* **36,** 192-236 (1974).

5. Kolaczyk, E. D. *Statistical Analysis of Network Data: Methods and Models.* (Springer, New York, 2009)

6. Hastie, T., Tibshirani, R. & Wainwright, M. *Statistical Learning with Sparsity: The Lasso and Generalizations* (CRC Press, New York, 2015)

7. Hastie, T., Tibshirani, R. & Friedman, J. *The Elements of Statistical Learning* (Springer, New York, 2001)

8. Wallis, W. D. *A Beginner's Guide to Graph Theory* (Birkhäuser, New York, 2007)

9. Little, R. J. A. Missing-data adjustments in large surveys. *J. Bus. Econ. Stat.* **6,** 287-296 (1988).

10. van Buuren, S. & Groothuis-Oudshoorn, K. Multivariate imputation by chained equations. *J. Stat. Softw.* **45,** 1-67 (2011).

11. Dalege, J. *et al.* Toward a formalized account of attitudes: The Causal Attitude Network (CAN) model. *Psychol. Rev.* **123,** 2-22 (2016).

12. Monroe, B. M. & Read, S. J. A general connectionist model of attitude structure and change: the ACS (Attitudes as Constraint Satisfaction) model. *Psychol. Rev.* **115,** 733-759 (2008).

13. Thompson, M. M., Zanna, M. P. & Griffin, D. W. in *Attitude Strength: Antecedents and Consequences* (eds. Petty, R. E. & Krosnick, J. A.) 361-386 (Lawrence Erlbaum, Hillsdale, 1995).

14. Fazio, R. H. in *Attitude Strength: Antecedents and Consequences* (eds. Petty, R. E. & Krosnick, J. A.) 247-282 (1995).

15. Zanna, M. P. & Rempel, J. K. in *The Psychology of Knowledge* (eds. Bar-Tal, D. & Kruglanski, A. W.) 315-334 (Cambridge Univ. Press, Cambridge, 1988).

16. Breckler, S. J. Empirical validation of affect, behavior, and cognition as distinct components of attitude. *J. Pers. Soc. Psychol.* **47,** 1191-1205 (1984).

17. van den Berg, H., Manstead, A. S. R., van der Pligt, J. & Wigboldus, D. H. J. The role of affect in attitudes toward organ donation and donor-relevant decisions. *Psychol. Health* **20,** 789-802 (2005).

18. Converse, P. E. in *The Quantitative Analysis of Social Problems* (ed. Tufte, E. R.) 168-189 (Addison-Wesley, 1970).

19. Achen, C. Mass political attitudes and the survey response. *Am. Polit. Sci. Rev.* **69,** 1218-1231 (1975).

20. Ansolabehere, S., Rodden, J. & Snyder, J. M. The strength of issues: Using multiple measures to gauge preference stability, ideological constraint, and issue voting. *Am. Polit. Sci. Rev.* **102,** 215-232 (2008).

21. Judd, C. & Milburn, M. The structure of attitude systems in the general public: Comparisons of a structural equation model. *Am. Sociol. Rev.* **45,** 627-643 (1980).
